# Skeletal Muscle Blood Flow and NIRS Oxygenation Kinetics as a Tool to Evaluate Adaptations to High-Intensity Exercise Training

**DOI:** 10.3390/s26103167

**Published:** 2026-05-16

**Authors:** Heru S. Lesmana, Patrick Rodrigues, Lydia L. Simpson, Kyohei Marume, Dean R. Perkins, Justin S. Lawley

**Affiliations:** 1 Department of Sport Science, University of Innsbruck, 6020 Innsbruck, Austria; herusl@fik.unp.ac.id (H.S.L.);; 2 Department of Sport Coaching, Universitas Negeri Padang, Padang 25132, Indonesia; 3 Division of Health, School of Sport and Human Movement, University of Waikato, Hamilton 3240, New Zealand; 4 School of Physiology, Pharmacology and Neuroscience, University of Bristol, Bristol BS8 1QU, UK; 5 Department of Cardiovascular, National Cerebral and Cardiovascular Center, Suita 564-0018, Japan; 6 Doctock Co., Ltd., Tokyo 107-0062, Japan; 7 Institute of Mountain Emergency Medicine, Eurac Research, 39100 Bolzano, Italy

**Keywords:** blood flow reactive hyperemia, NIRS muscle oxygenation, NIRS post-exercise, high-intensity interval training

## Abstract

Exercise training improves maximum aerobic capacity, in part, through improvements in skeletal muscle function. This study aimed to investigate adaptations to improved aerobic capacity training through non-invasive and non-exhaustive tests of hyperemic muscle blood flow and near-infrared spectroscopy (NIRS) muscle oxygenation kinetics. An experimental study was conducted on 18 participants (age, 28.2 ± 5.3 yr; _abs_*V*O_2max,_ 3.60 ± 0.67 L·min^−1^). Before and after the intervention of a 6-week of high-intensity interval training (HIIT), participants underwent three tests: (1) a graded cardiopulmonary exercise test; (2) a vascular occlusion test; and (3) a steady-state exercise (SSE) at 60% of PPO. Expired gas analysis, superficial femoral blood flow (occlusion test only) and SmO_2_ kinetics were measured. The intervention increased maximal aerobic capacity _abs_*V*O_2max_ (*p* < 0.001, *d* = 0.65) and PPO (*p* < 0.001; *d* = 0.41). Moreover, steady-state _abs_*V*O_2_ (*p* = 0.006; *d* = 0.37) and HR (*p* = 0.001; *d* = 0.65) were reduced. With the cuff test, the SmO_2_ desaturation slope increased (*p* = 0.04; *d* = 0.52), while peak muscle blood flow (*p* = 0.02; *d* = 0.51) and the SmO_2_ 10 s reoxygenation rate increased (*p* < 0.001 *d* = 1.11; 0.74 ± 0.28 to 1.17 ± 0.45%/s). During steady-state exercise, SmO_2_ decreased less (*p* = 0.02; *d* = 0.43), and the 10s recovery kinetics rate was slowed (*p* = 0.01 *d* = 0.30; 0.28 ± 0.20 to 0.22 ± 0.21%/s). The improvement in *V*O_2max_ had a moderate correlation with the SmO_2_ recovery rate post-steady-state exercise (*p* = 0.05, r = −0.54). HIIT changed maximal aerobic capacity alongside improvements in skeletal muscle hyperemic blood flow, SmO_2_ post-occlusive reactive hyperemia and SmO_2_ post-exercise recovery kinetics. Thus, the findings indicated that non-invasive and non-exhaustive hemodynamic kinetic profiles can monitor adaptations to improved aerobic capacity.

## 1. Introduction

Regular exercise yields many physiological benefits, including improved musculoskeletal function, enhanced cardiovascular capacity, and overall health [1]. For athletes, structured training programs are essential to optimize cardiovascular fitness and performance. The classic test of aerobic fitness and training efficacy is the maximal cardiopulmonary exercise test and quantification of maximal oxygen consumption (*V*O_2max_). However, the *V*O_2max_ test has three key limitations [2]: (1) it requires maximal exercise for accurate results, (2) it is costly and demands specialized equipment and personnel, and (3) it cannot distinguish between central and peripheral adaptations. The final limitation is particularly important.

Central adaptations include increased cardiac output, plasma volume, and red (blood) cell mass, all of which enhance oxygen transport [3,4]. In contrast, peripheral adaptations involve improvements in capillary density, vascular function, and mitochondrial content and function [3,5]. During cardiopulmonary exercise testing, gas exchange is measured at the mouth. However, oxygen transport from the air to the mitochondria occurs in a series of steps, which are not measured individually by indirect calorimetry, but are rather integrated into a single value. This situation has prompted growing interest in alternative and/or supplementary methods to assess training adaptations, particularly at the skeletal muscle level.

Commercial near-infrared spectroscopy (NIRS) devices have become increasingly compact, powerful, and accessible, emerging as a promising non-invasive, wearable technology. Wearable NIRS continuously measures the ratio of hemo-/myoglobin to hemo-/myoglobin and myoglobin (SmO_2_ = ([HbO_2_]/[HbO_2_] + [HHb]) × 100), yielding real-time muscle oxygen saturation (SmO_2_) [6,7]. Over the past decade, several NIRS-based approaches have shown utility in assessing skeletal muscle function [7]. These include limb ischemia with post-ischemic reactive hyperemia [8,9,10,11] and post-exercise NIRS recovery kinetics [12,13]. We have previously shown that both methods can predict an individual’s *V*O_2max_ [14]. Moreover, although NIRS does not directly quantify muscle blood flow, kinetic profiles from non-exhaustive limb ischemia tests are equally predictive of *V*O_2max_ as an advanced ultrasound-based assessment of muscle hyperemia [15]. One key limitation of prior studies is their correlational design. Since *V*O_2max_ reflects both central and peripheral components of convective and diffusive oxygen transport, it is expected that a measure of peripheral vascular function correlates with *V*O_2max_. However, for monitoring training adaptations, the critical factor is whether these methods are sensitive enough to detect changes induced by structured training. Several studies have documented that high-intensity interval training (HIIT) can improve *V*O_2max_ [16] alongside both central [3] and peripheral adaptions in the oxygen cascade [17]. Indeed, a recent study has observed an increase in peripheral vascular function (assessed via ultrasonography) post-6 weeks of exercise training [18]. Thus, the aim of this study was to detect training-induced improvements in skeletal muscle function through non-exhaustive tests of vascular function via the gold standard approach of ultrasonography complemented with SmO_2_ recovery kinetics via NIRS-based technologies following six weeks of HIIT. Importantly, NIRS is becoming a popular tool amongst athletes in applied settings where sensor placement is not standardized. To enhance external validity, we used a simplified procedure in which participants flexed the Vastus Lateralis and the sensor was placed over its most prominent region. Although less precise than typical mechanistic approaches, this method better reflects real-world use and is therefore critical for determining whether these sensors provide accurate information under applied conditions.

## 2. Materials and Methods

### 2.1. Participants

This study enrolled 18 healthy participants, including 10 males (age, 29.6 ± 6.5 yr; stature, 1.82 ± 5.7 m; body mass, 85.1 ± 10.0 kg; blood pressure, 131/84 ± 12/8 mmHg; baseline _abs_*V*O_2max_, 3.97 ± 0.64 L·min^−1^; baseline _rel_*V*O_2max_, 46.5 ± 10.7 mL·kg·min^−1^) and eight females (age, 26.0 ± 1.6 yr; height, 1.70 ± 4.4 m; weight, 62.8 ± 2.5 kg; blood pressure, 130/82 ± 10/7 mmHg; baseline _abs_*V*O_2max_, 3.14 ± 0.44 L·min^−1^; baseline _rel_*V*O_2max_, 48.9 ± 7.35 mL·kg·min^−1^). All were students at the University of Innsbruck, generally fit (Table 1), and free from cardiovascular, respiratory, or metabolic disease. Participants were not taking medications affecting hemodynamic responses to exercise. Two were excluded due to an unrelated muscle injury and flu symptoms. Written informed consent was obtained following detailed verbal explanation of all experimental procedures and risks. The study adhered to the Declaration of Helsinki, except database registration, and was approved by the University of Innsbruck ethics committee (No. 101/2023).

### 2.2. Protocol

**Figure 1:**
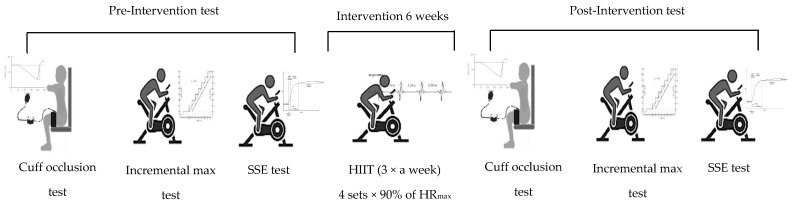
Schematic of experimental design in this study. Cuff occlusion test with 300 mmHg pressure for 3 min; incremental max test started at 100 W, with 5 W increments every 15 s until volitional exhaustion or inability to maintain ≥85 rpm; SSE test with cycling at 60% of PPO for 3 min. SSE = steady-state exercise; HIIT = high-intensity interval training; HR_max_ = maximum heart rate.

Pre- and post-intervention testing was conducted over two visits on consecutive days. Participants abstained from alcohol, caffeine, smoking, and exercise for 12 h and consumed a light meal four hours prior to testing. During the first visit, body mass, stature, and blood pressure were measured, followed by the cuff occlusion test and lastly, an incremental maximal exercise test. On the second visit, participants completed the steady-state exercise test (SSE) (Figure 1). All tests were conducted at the same time of day (±30 min). Female participants were tested during the same menstrual phase at both time points. Testing sessions were conducted approximately eight weeks apart (after 6 weeks intervention), and all female participants reported regular cycles ranging from 28 to 35 days.

#### 2.2.1. Cuff Occlusion Test

Participants sat on a padded chair with their lower leg hanging. A thigh pressure cuff (Hokanson CC17, Bellevue, WA, USA; sized 18 cm × 108 cm) was placed at the inguinal crease of the right upper thigh regardless of leg dominance and connected to a blood pressure monitor (Heinie Gamma M-00.09.240, Herrsching, Germany). After 2 min rest, the thigh cuff was inflated to 300 mmHg for 3 min. Participants relaxed the leg during inflation and for 3 min after a rapid deflation.

#### 2.2.2. Incremental Maximal Exercise Test

The test was conducted in a quiet room (~25 °C). A spirometric system was utilized to record ventilation (Pneumotach amplifier 1 series 1110, Hans Rudolph Inc., Shawnee, KS, USA) and expired gases (gas analyzer ML206, ADInstruments Pty Ltd., Bella Vista, NSW, Australia) continuously thru a mixing chamber (MLA246, ADInstruments Pty Ltd., Bella Vista, NSW, Australia) and was calibrated prior to each measurement. A Bluetooth chest belt (Wear Link; Polar, Kempele, Finland) was attached to monitor heart rate (HR) and transmitted to the spirometric device. Participants cycled on an ergometer (Cyclus 2, Leipzig, Germany) starting at 100 W, with 5 W increments every 15 s until volitional exhaustion. Participants were allowed to choose their preferred cadence between 90 and 120 rpm but were requested to maintain it at ±5 rpm once their preferred cadence was achieved, for the entire test.

#### 2.2.3. Steady-State Exercise Test

The same gas analyzer, HR monitor and cycling ergometer detailed above were used for the steady-state exercise test. Following a 3 min warm-up at 40% of peak power output (PPO), participants rested for 5 min, then cycled at 60% of PPO for 3 min. They then stopped pedaling immediately and maintained the right leg in a relaxed neutral position, with their feet kept clipped into the pedal and the leg extended fully (straight down 180°) for 3 min. Based on our previous study, 3 min of exercise was an adequate duration to reach steady-state SmO_2_ and showed the recovery kinetic correlated to *V*O_2max_ [14]. The power output remained fixed for pre- and post-tests.

### 2.3. Interventions

This study was part of a larger protocol investigating the effects of passive hot-water immersion on performance. All participants completed a 6-week cycling HIIT program consisting of 4 min intervals at 90% of maximal heart rate, separated by 3 min rest or active recovery, repeated four times. Heart rate was monitored during all sessions using the Polar Beat app to ensure adherence to training zones. Participants also underwent progressive hot-water (42 ± 0.3 °C; N = 9) or thermoneutral immersion (34.5 ± 0.2 °C; N = 9) 5 days per week. Immersion time progressed from 40 min (weeks 1–2) to 50 min (weeks 5–6). Water was consumed ad libitum, and fans were used to reduce dehydration and increase comfort. All participants completed 18 supervised HIIT sessions and between 26 and 30 water immersion sessions. The effect of passive hot-water immersion has been reported previously by our group [19]. The primary outcome was to assess whether non-exhaustive measures of skeletal muscle function could detect training-induced adaptations. As both groups completed identical HIIT, data were pooled for analysis. All participants were permitted to keep their regular physical activities (e.g., strength training, CrossFit, jogging, hiking, horse riding, handball, volleyball, football, skiing, snowboarding and yoga) and maintain the usual level of activity from the previous 2 months. They also were asked to refrain from any new activities involving environmental stress (e.g., sauna, cold-water immersion, altitude training). To monitor the adherence of participants to the training volume throughout the intervention period, participants completed a report at the conclusion of each week that included all exercises, activities, and volumes completed outside of the study.

### 2.4. Measurements

#### 2.4.1. Superficial Femoral Artery Blood Flow

Superficial femoral artery blood flow (sFBF) velocity was measured at an insonation angle of 60° alongside artery diameter using a 15–4 Mhz transducer (15L4 Smart MarKᵀᴹ, Terason, Burlington, MA, USA) via Duplex ultrasonography (Usmart 3300 NexGen Ultrasound; Teratech Corporation, Burlington, MA, USA). The intensity weighted time-averaged mean blood velocity was measured with the sample volume encompassing the entire vessel lumen. Continuous measurements were made using a Camtasia Recorder (Camtasia Recorder 8; TechSmith, East Lansing, MI, USA) and analyzed offline with custom software (version 29-Aug-2024). An experienced sonographer conducted all measurements, with probe placement marked on the skin for consistency.

#### 2.4.2. Skeletal Muscle Oxygenation Through Near-Infrared Spectroscopy

**Figure 2:**
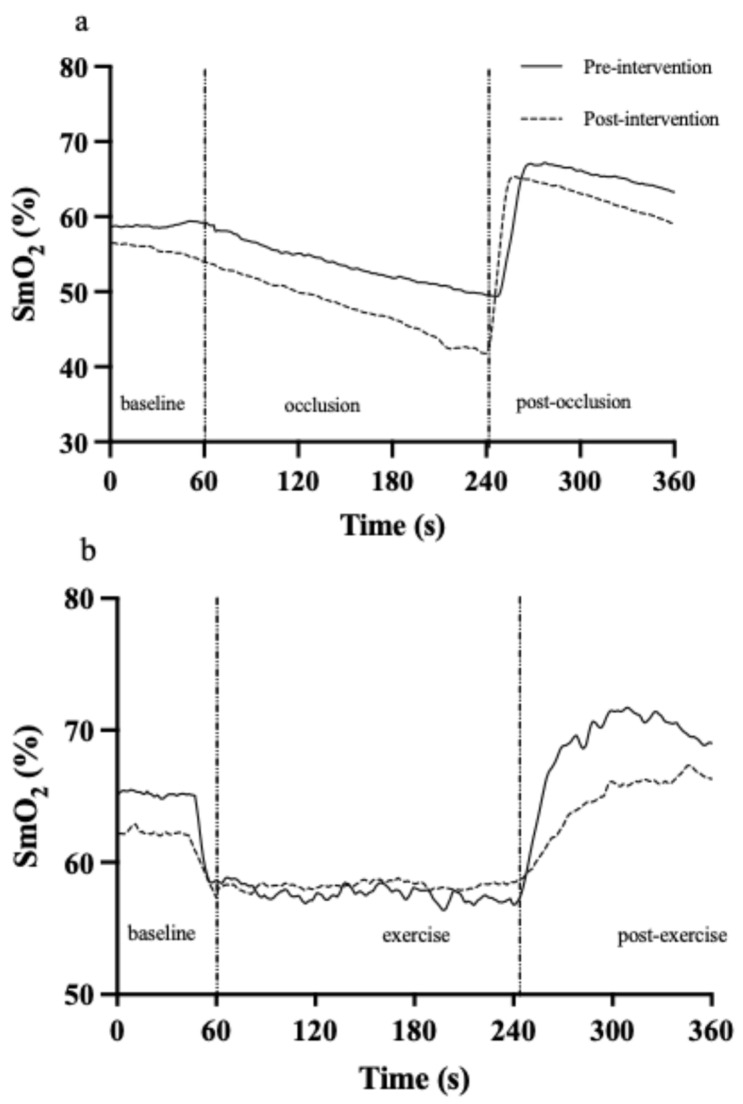
Representative changes in muscle reoxygenation (SmO_2_) pre- and post-intervention during (**a**) cuff occlusion test and (**b**) steady-state exercise.

A commercial near-infrared spectroscopy (NIRS) sensor (Train.Red Fyer, Train.Red Manufactures, Gelderland, The Netherlands) was attached to the most prominent part of the Vastus Lateralis for the cuff occlusion test and SSE test. Prior studies have shown this device provides stable, precise SmO_2_ estimates during in vivo and vascular occlusion tests [20,21]. SmO_2_ (oxy-hemoglobin/total-hemoglobin × 100) was measured continuously at a sampling frequency of 10 Hz with the method of Spatial-Resolved Spectroscopy. The sensor uses two wavelengths (760 and 850 nm) with a light source and detector distance of 35 mm and a measurement depth ~20 mm. The sensor was covered with “blackout” fabric and a bandage to prevent light artifacts and ensure stability (for representative data see Figure 2).

### 2.5. Data Analysis

#### 2.5.1. Maximal Aerobic Capacity

An analog-to-digital converter (Powerlab: ADInstruments, Oxford, UK) recorded ventilation, gas exchanges and HR data during the incremental and SSE test. Data were displayed on LabChart (LabChart 8; ADInstruments, Oxford, UK) and analyzed offline. Gas exchange and HR were averaged over 30 s. Absolute *V*O_2max_ (_abs_*V*O_2max_) was confirmed as a plateau in oxygen uptake and accepted as the highest 30 s average. Maximal heart rate (HR_max_) and peak power output (PPO) were recorded at the corresponding timepoint. Relative *V*O_2max_ (_rel_*V*O_2max_) was calculated as *V*O_2max_ divided by body weight [22]. SSE test _abs_*V*O_2_ and HR were taken as the last 30 s average.

#### 2.5.2. Reactive Hyperemia

Baseline artery diameter and blood velocity were measured 1 min prior to cuff inflation and continuously for 3 min post-occlusion [15]. Femoral blood flow was determined using established methods [22,23]. Post-occlusion reactive hyperemia and microvascular dilator function were assessed via (1) peak blood flow, defined as the highest averaged value across three consecutive heartbeats post-release of the cuff, and (2) the difference in blood flow between baseline and peak flow (ΔsFBF baseline–peak) to correct for any potential differences in baseline blood flow between individuals or sessions. One case was excluded due to delayed insonation after cuff release, where peak flow would have been missed.

#### 2.5.3. NIRS Reoxygenation

SmO_2_ data were exported to LabChart for offline analysis. Baseline SmO_2_ was averaged over the last 30 s before the cuff occlusion test and before the SSE test. Post-cuff release and post-exercise reoxygenation metrics included the 10 s reoxygenation rate (Rep 10 s) and rate of reoxygenation to peak (Rpeak). Moreover, the rate of reoxygenation back to baseline was also calculated post-SSE (SSE Rbl). The increment or delta oxygenation (I) and time constant of muscle reoxygenation (τ) were calculated by multiplying 0.63. Subsequently, the relative muscle reoxygenation rate (R) was calculated: R = I/τ [24,25]. Therefore, R_peak_ was calculated with (I_peak_/τ_peak_) and SSE R_bl_ (SSE I_bl_/SSE τ_bl_). During the cuff test, the linear slope of the SmO_2_ signal s was also quantified over the entire occlusion period using the liner slope formula in LabChart. For a detailed visual of the time course of SmO_2_ during these tests, see Figure 2, and for quantification timings see [15]. Data were excluded (*n* = 5) if the NIRS signal was lost post-cuff release (cuff occlusion test) or (*n* = 4) during the exercise and post-exercise period (SSE test).

### 2.6. Statistical Analysis

Data are presented as mean ± standard deviation (SD), with significance set at *p* < 0.05. Pre- vs. post-intervention changes in aerobic capacity, post-exercise and post-occlusion reoxygenation, and reactive hyperemia were analyzed using Paired T-tests. Effect sizes (*d*) were calculated using Cohen’s d. Post hoc Pearson’s correlations examined relationships between changes in *V*O_2max_ and changes in reactive hyperemia peak flow, muscle reoxygenation, and occlusion slope, with coefficients classified per recommendations. Analyses were performed using SPSS (Version 29, IBM, Chicago, IL, USA), and the graphs were created on GraphPad Prism (Version 9.1.1, GraphPad Software, La Jolla, CA, USA).

## 3. Results

### 3.1. Changes in Classic Parameters Associated with Exercise Training

**Figure 3:**
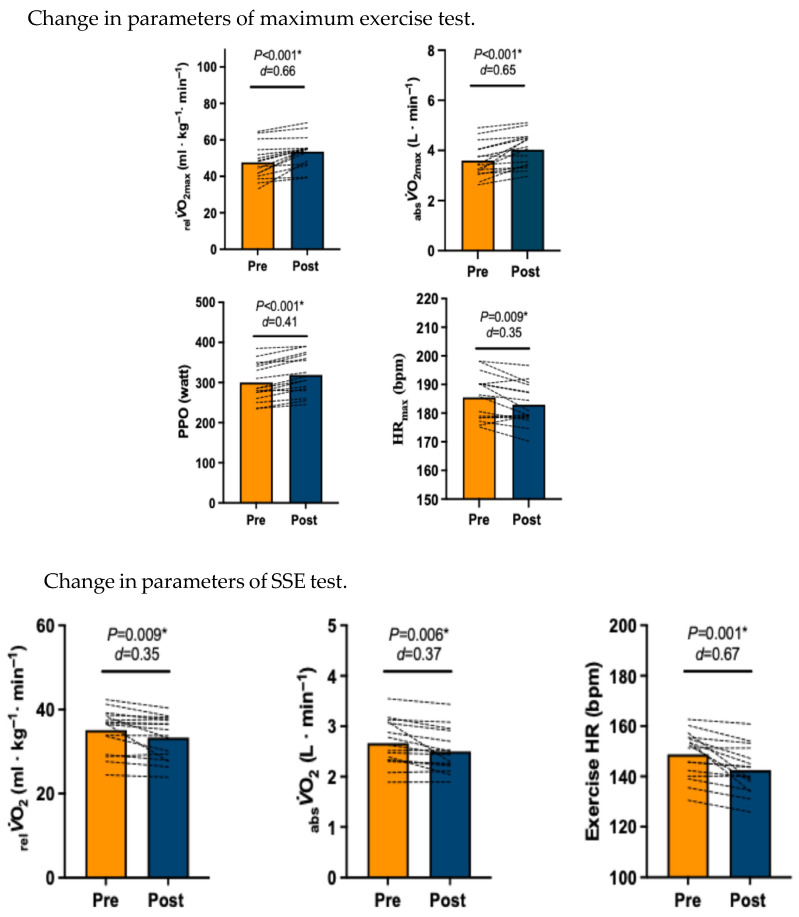
Changes in parameters of maximal exercise and steady-state exercise pre- and post-6 weeks of high-intensity interval training (HIIT). (**Upper Panel**). Relative (_rel_*V*O_2max_) and absolute *V*O_2max_ (_absl_*V*O_2max_), peak power output (PPO) and maximum heart rate (HR_max_) for the maximal exercise (*n* = 16) (**lower panel**). *V*O_2_ for relative (_rel_*V*O_2_) and absolute (_absl_*V*O_2_) exercise heart rate when the steady state is reached. The asterisk (*) indicated a significant different.

Six weeks of HIIT moderately increased _rel_*V*O_2max_ (*p* < 0.001, *d* = 0.66; 47.69 ± 9.49 to 53.56 ± 8.29 mL·kg·min^−1^) and _abs_*V*O_2max_ (*p* < 0.001, *d* = 0.65; 3.60 ± 0.67 to 4.03 ± 0.66 L·min^−1^). PPO increased (*p* < 0.001, *d* = 0.41; 300 ± 47.1 to 320 ± 48.9 watt) after the intervention and HR_max_ showed a decrease (*p* = 0.009, *d* = 0.35; 186 ± 7.92 to 183 ± 7.11 bpm) (see Table 2). In the SSE test, _abs_*V*O_2_ (*p* = 0.006, *d* = 0.37; 2.66 ± 0.46 to 2.50 ± 0.43 L·min^−1^) and _rel_*V*O_2_ (*p* = 0.009, *d* = 0.35; 35.1 ± 5.13 to 33.3 ± 5.07 mL·kg·min^−1^) decreased, highlighting greater efficiency. Mean exercise HR (*p* = 0.001, *d* =0.65; 149 ± 9.29 to 142 ± 9.25 bpm) during the SSE showed a moderate decline (see Table 3 and Figure 3).

### 3.2. Changes in Superficial Femoral Blood Flow

**Figure 4:**
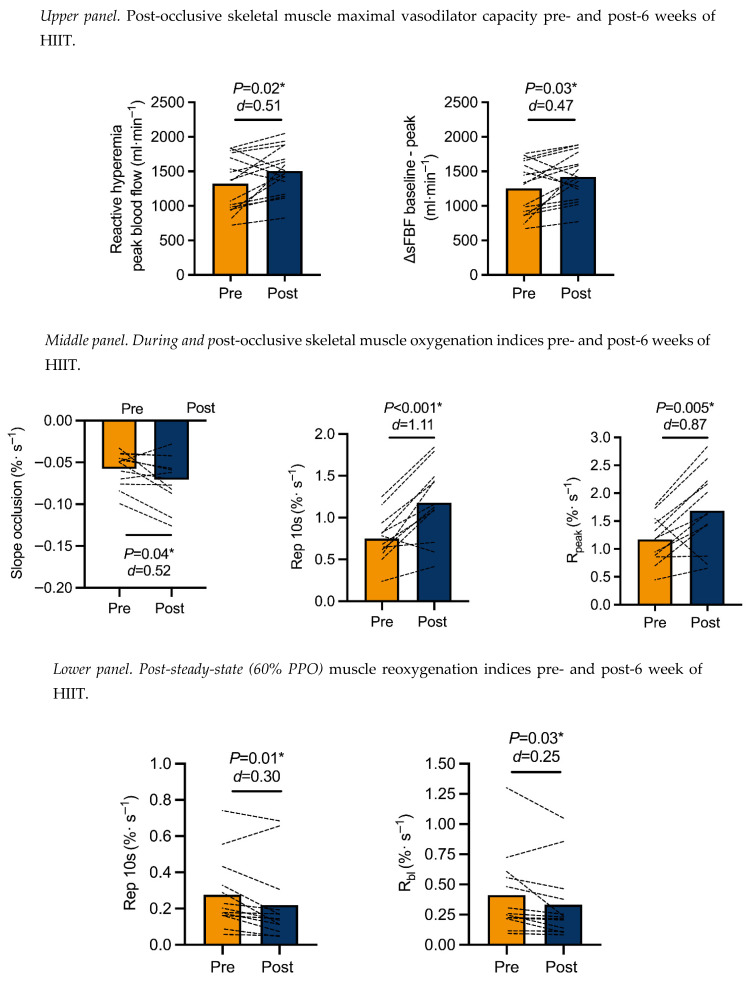
Changes in ultrasound and near-infrared spectroscopy-based metrics of skeletal muscle pre- and post-6 weeks of high-intensity interval training (HIIT) (**upper panel**). Peak superficial femoral artery blood flow after 3 min of occlusion (300 mmHg) (*n* = 16) (**middle panel**). The oxygenation occlusion slope and two indices of muscle reoxygenation (*n* = 12), i.e., the muscle reoxygenation rate over 10 s (Rep 10 s) and the relative muscle oxygenation to peak (R_Peak_) (**lower panel**). Two indices of muscle reoxygenation post-3 min of cycling at 60% of peak power output (*n* = 13), i.e., Rep 10 s, reperfusion rate over 10 s; R_bl_, relative rate of muscle reoxygenation back to baseline. The asterisk (*) indicated a significant different.

Reactive hyperemia peak flow (*p* = 0.02; *d* = 0.51; 1253 ± 337 to 1494 ± 290 mL·min^−1^) and ΔsFBF baseline–peak (*p* = 0.03; *d* = 0.47; 1253 ± 384 to 1422 ± 334 mL·min^−1^) increased (Figure 4).

### 3.3. Changes in Muscle Reoxygenation During and Post-Cuff Occlusion

SmO_2_ Rep 10 s (*p* < 0.001 *d* = 1.11; 0.74 ± 0.28 to 1.17 ± 0.45%/s) and R_peak_ (*p* = 0.005 *d* = 0.87; 1.17 ± 0.42 to 1.69 ± 0.71%/s) post-occlusive reactive hyperemia improved post-six weeks of HIIT, while the SmO_2_ occlusion slope became steeper (*p* = 0.04 *d* = 0.52; −0.06 ± 0.02 to −0.07 ± 0.03%/s) (Figure 4).

### 3.4. Changes in Muscle Reoxygenation Post-Steady-State Exercise at an Absolute Workload

Baseline SmO_2_ was not different (*p* = 0.25 *d* = 0.02; 68.2 ± 5.03 to 67.2 ± 5.71%), but pre-recovery SmO_2_ decreased after HIIT (*p* = 0.02 *d* = 0.43; 60.4 ± 7.05 to 63.4 ± 6.93%). On cessation of SSE, the Rep 10 s (*p* = 0.01 *d* = 0.30; 0.29 ± 0.23 to 0.23 ± 0.21%/s) and SSE R_bl_ (*p* = 0.03 *d* = 0.25; 0.43 ± 0.34 to 0.34 ± 0.31%/s) (Figure 4) were reduced. This was followed by the reduction in SSE I_bl_ (*p* = 0.01 *d* = 0.46; 5.87 ± 4.36 to 4.05 ± 3.41%) and SSE τ_bl_ (*p* = 0.01 *d* = 0.73; 14.5 ± 3.38 to 12.2 ± 3.09 s).

### 3.5. Relationships Between the Change in Absolute VO_2max_ (_abs_VO_2max_) and Change in Blood Flow and NIRS Kinetics

**Figure 5:**
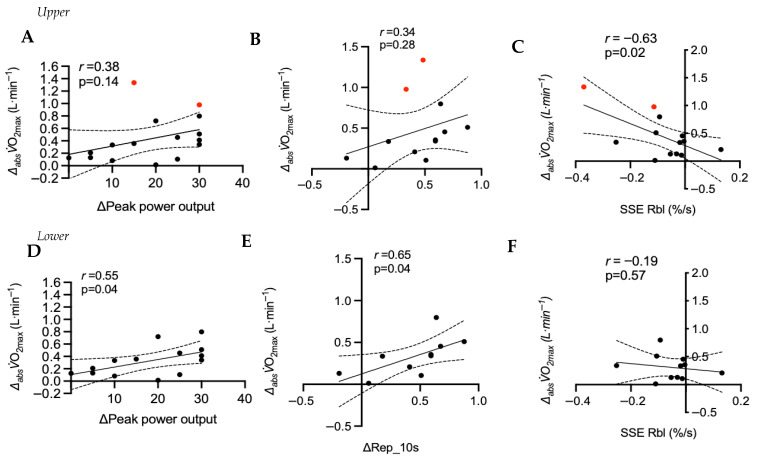
(**Upper panel**) Linear correlation between change in absolute *V*O_2max_ (_absl_*V*O_2max_) and change in (**A**) peak power output (*n* = 16) and cuff-based metrics of (**B**) reperfusion rate of reoxygenation over 10 s (Rep 10 s) and (**C**) post-exercise SmO_2_ recovery time to baseline. (**Lower panel**) Same linear correlations, but after two individuals (red dot) with major improvements (~1 L·min^−1^) in *V*O_2max_ have been removed (**D**–**F**). SSE, steady-state exercise; R_bl_, relative rate of muscle reoxygenation back to baseline. The dotted line represents the 95% confidence interval of the model.

The change in _abs_*V*O_2max_ was not statistically related to the change in reactive hyperemia peak flow (r = 0.34, *p* = 0.28), or the Rep 10 s post-occlusive reactive hyperemia (r = 0.37, *p* = 0.24). A negative non-statistically significant relationship was measured between the change in _abs_*V*O_2max_ and post-SSE Rep 10 s (r = −53, *p* = 0.06), whereas R_bl_ post-exercise recovery kinetics had a negative moderate correlation (r = −0.63, *p* = 0.02) (Figure 5).

## 4. Discussion

This study investigated whether adaptations to improved aerobic capacity could be detected using non-exhaustive assessments of muscle blood flow and oxygen kinetics during and after leg ischemia and steady-state exercise. The main findings were that classic aerobic fitness markers improved, with increased PPO and *V*O_2max_, reduced HRmax, and lower steady-state *V*O_2_ and HR at a fixed workload. Training intervention in the present study increased peak femoral artery blood flow (reactive hyperemia) following 3 min of leg ischemia, likely connected to the steeper deoxygenation slope during cuff inflation [9,15,26], and accelerated both reoxygenation rate and peak reoxygenation post-cuff release. During steady-state exercise, minimum SmO_2_ was attenuated, while post-exercise reoxygenation was slower after 6 weeks of HIIT.

### 4.1. Improved Aerobic Capacity and Muscle Blood Flow and NIRS Oxygenation Kinetics During and Post-Ischemic Limb Occlusion

These findings align with previous reports showing improvements in absolute and relative *V*O_2max_, peak power output, and reduced maximal HR following HIIT [1,3,27]. The present study also confirms findings from a recent study [18] that HIIT leads to an increase in peak femoral artery blood flow (reactive hyperemia) after 3 min of leg ischemia, consistent with the concept that endurance training enhances skeletal muscle vasodilator function [28,29]. Yet interpreting this response requires consideration of the relationship between SmO_2_ during occlusion (i.e., O_2_ debt) and the magnitude of hyperemia. Previous work by Rosenberry and Nelson [29], confirmed by our group [15], indicates that peak blood flow post-ischemia is largely driven by the magnitude of hypoxia during cuff inflation. In the current study, improved aerobic capacity increased the deoxygenation slope (and the integral or area under the curve) during cuff occlusion, suggesting a stronger hypoxic stimulus for downstream vasodilation [30], which should lead to greater peak blood flow and faster reoxygenation kinetic [15]. Thus, the observed improvement in post-ischemic reactive hyperemia may not reflect an improvement in vasodilator function per se. Interestingly, the cause of the greater deoxygenation rate during cuff occlusion with improved aerobic capacity is not entirely clear. While the occlusion test with NIRS is often used to assess resting muscle metabolism [7,31], substantial gains in leg muscle mass over 6 weeks are unlikely [5]. Therefore, the greater deoxygenation slope may represent improved mitochondrial content and oxidative function and faster O_2_ utilization during ischemia [3,32,33]. Irrespective of the underlying mechanism(s), the deoxygenation slope is a modest predictor of fitness [14], and more importantly, the current study seems to suggest that either the deoxygenation slope or the reactive hyperemia is a sensitive metric for estimating changes in skeletal muscle function with HIIT.

### 4.2. Improved Aerobic Capacity and NIRS Post-Steady-State Oxygenation Recovery Kinetics

NIRS reoxygenation indices are quicker post-steady-state exercise in fit individuals [14]. This is likely because, if the steady-state workload is performed relative to the individual’s *V*O_2max_, then the absolute workload and *V*O_2_ will be higher, the degree of exercising tissue hypoxia will be greater, and thus a greater signal for skeletal muscle vasodilation causes faster reoxygenation kinetics. HIIT reduced the oxygen cost and HR response during steady-state exercise at a fixed absolute workload equivalent to 60% of pre-training PPO. This aligns with prior studies reporting improved oxygen efficiency following endurance training [1,27,34]. After steady-state exercise with 60% PPO, post-exercise reoxygenation indices were slower following improved aerobic capacity. While this appears to contrast with the faster reoxygenation rate observed in fit individuals and post-ischemic cuff occlusion (i.e., near maximal tissue hypoxia for the current state of the skeletal muscle Figure 2), it aligns with the aforementioned relationship between the reduction in SmO_2_ and hyperemia. After 6 weeks of HIIT, the oxygen cost of steady-state exercise at a fixed absolute workload decreased, reflected by an attenuated *V*O_2_ and a smaller drop in skeletal muscle SmO_2_ during steady-state exercise (60% ± 7 to 63% ± 7, *p* = 0.018). Therefore, the stimulus for skeletal muscle vasodilation (i.e., downstream vascular resistance) is reduced due to the diminished need for muscle blood flow (i.e., metabolic (*V*O_2_)-flow matching). Consequently, when exercise stops abruptly, recovery kinetics slow due to a relative reduction in downstream vascular conductance. A similar attenuated fall in SmO_2_ during steady-state workloads has been observed in kayakers post-training after nine HIIT sessions [35]. One study utilizing the multiple cuff occlusion technique to estimate post-exercise skeletal muscle oxidative capacity observed an improvement with 6 weeks of wrist flexor endurance training [36]. However, in contrast to our findings, Barberan et al. [20] found greater NIRS recovery kinetics post-steady-state cycling following 8 weeks of moderate-intensity endurance training in elderly patients with chronic obstructive pulmonary disease and healthy age match controls [20]. These divergent findings are difficult to reconcile. While differences in training modality (moderate vs. high intensity) may contribute, variations in trainability due to age (65 ± 11 vs. 28 ± 5.3 yrs) and baseline fitness level (_abs_*V*O_2max_: 1566 ± 410 vs. 3600 ± 67 mL∙min^−1^) are more plausible explanations and warrant further investigation.

### 4.3. Relationship Between Improvements in Skeletal Muscle Function and VO_2max_

Endurance training improves central [3,4] and peripheral factors [3]. While findings are not uniform [37], HIIT is particularly adept at enhancing peripheral adaptations to exercise [1]. Thus, if skeletal muscle NIRS profiles reflect important underlying adaptions in the muscle, a relationship with improvements in *V*O_2max_ would be expected. This would align with previous findings using non-invasive measures of skeletal muscle function from NIRS technology [8,9,10,31,38]. While the current study included a relatively small sample for correlational analyses, a modest negative relationship was observed (r = −0.63, *p* = 0.02) between the change in the NIRS recovery rate post-steady-state exercise and the improvement in *V*O_2max_. However, caution is warranted with a notable outlier. For the ischemic cuff test, relationships were less clear. Two individuals showed large *V*O_2max_ increases (~1 L∙min^−1^), possibly due to 1) technical measurement error or 2) major central adaptations in stroke volume or hematological parameters. Excluding these individuals revealed a stronger correlation between NIRS-derived peripheral adaptations and *V*O_2max_, while the relationship with post-exercise NIRS recovery kinetics disappeared (Figure 5). While caution is warranted due to the subjectivity of outlier removal, this approach is supported by an improved and expected relationship between peak power output and *V*O_2max_ (Figure 5, lower panel A) and between peripheral adaptations and *V*O_2max_. Notably, a prior study in youth cyclists reported a strong correlation between improvements in *V*O_2max_ and muscle deoxygenation responses during incremental exercise with 3 years of training [39].

### 4.4. Practical Implications

The main practical implications from this study are that peripheral skeletal muscle adaptations to improved aerobic capacity can be monitored using non-exhaustive, non-invasive methods. Given the technical demands of ultrasonography, its routine use in athlete monitoring is limited. In contrast, NIRS is cost-effective and user-friendly in both probe placement and data processing. From a practical standpoint, classic cardiopulmonary exercise testing can be supplemented with the proposed NIRS-based protocols. The ischemic cuff test, in particular, is low-cost and can be performed frequently, while the moderate exercise intensities used here could be integrated into warm-up routines. Together, these methods offer a feasible approach for continuous monitoring between less frequent maximal tests. Although this study does not define optimal testing or post-processing protocols, the procedures employed were reliable and sensitive enough to be detect after improving aerobic capacity. Notably, classic research controls, such as adipose tissue thickness, total hemoglobin normalization, and precise sensor placement, were not strictly followed. Instead, the NIRS sensor was placed using a standardized procedure by having the participant flex the Vastus Lateralis and positioning the sensor over its most prominent region (see Section 4.5). Thus, while precise sensor placement should remain the standard approach in research studies using NIRS to examine underlying physiological mechanism, these experimental data, alongside our two validations studies using the same approach [14,15], suggest that this simplified approach may be applicable to the broader athletic population beyond the research community. Future refinements will likely establish these techniques as routine metrics for endurance training assessment.

### 4.5. Limitations

A clear limitation is the exclusion of a non-exercise control group and, as such, we cannot conclusively attribute improvement in cardiorespiratory and NIRS parameters to HIIT alone. Indeed, theoretically, improvements in *V*O_2max_, steady-state variables, ultrasound and NIRS-based metrics could have all been due to measurement error and biological variability. While we acknowledge these limitations, measurement error and biological variably are highly unlikely because they would require a unidirectional measurement error in several independent techniques (gas analysis, heart rate, ultrasonography, and NIRS). While this is highly unlikely, it is acknowledged as a limitation. Finally, even if improvements in *V*O_2max_ resulted from systemic lifestyle modifications independent of HIIT, the primary aim of this study was to determine whether changes in skeletal muscle function could be detected alongside increases in *V*O_2max_. Regardless of the underlying mechanisms, our findings demonstrate that aerobic fitness improves alongside improvements in ultrasound and NIRS-derived metrics of skeletal muscle function.

Another limitation is that we did not assess adipose or skin thickness, and the NRIS sensor placed followed a more simplified standardization procedure. Sensor placement is known to affect the NIRS signal, and the depth of infrared penetration can affect absolute NIRS units [21], particularly given the known variability in thigh adiposity [36]. While these are limitations, they are also both strengths of the study, as this approach substantially improves the external validity of our results. Firstly, we previously validated this approach in two studies [14,15] whereby NRIS recovery kinetics were related to *V*O_2max_ despite this simplified procedure. Secondly, this study observed improvements in skeletal muscle function via ultrasonography, which is the gold standard approach. The NIRS metrics mirrored these findings with large effect sizes and a high level of statistical significance. Thus, although the simplified standardization procedure likely increased variability in the NIRS data, our results show that meaningful changes can still be detected with a simplified placement procedure, particularly when effect sizes are large following several weeks of HIIT. Finally, it is worth mentioning the potential outlier in the correlational analysis. While we felt this information was interesting to present to the reader, it represents a post hoc tertiary analysis. While, in our opinion, it is interesting that some NIRS metrics may be sensitive to the within-person improvements in *V*O_2max_, these correlational data are limited, and we present both datasets for transparency. Nonetheless, these correlational analyses should not distract from the primary experimental nature of this study. Another potential drawback is that the pre- and post-SSE were performed at an absolute workload relative to the initial *V*O_2max_. However, this is important for the applied nature and external validity of the current study because the aim was to identify if steady-state NIRS recovery kinetics could monitor improvements with HIIT without having to perform repeated maximal exercise tests to normalize workloads. The next limitation is that the current study only included individuals who were generally fit. Future research is warranted to identify if similar metrics can identify small improvements in clinical and elite populations. Finally, the primary aim of this study was to identify if improvements in non-exhaustive tests of the skeletal muscle could be observed with structured training and, as such, we used an opportune sample consisting of two groups performing 6 weeks of HIIT, but one experimental group also performed 5 days of passive hot-water immersion each week [19]. From our perspective, this does not affect the interpretation of our data because our goal was only to improve *V*O_2max_ and observe if skeletal muscle function improved similarly. Nonetheless, hot-water immersion is known to influence vascular function independent of training and to augment training adaptations in this sample [19] and others [40]. Thus, it would be interesting to see if these non-exhaustive ultrasounds and NIRS-based tests are also impacted by the supplementation of passive hot-water immersion. Firstly, it is unlikely that the improvements in these metrics were due to the addition of passive heating alone because improvements were related to changes in *V*O_2max_ and, at least for NIRS recovery kinetics, were present in the HIIT-only group that did not perform passive hot-water immersion (see Supplementary Materials). Secondly, while this study was not statistically powered for that comparison, these data have been provided in the Supplementary Materials. While cautious interpretation is obviously warranted, it is interesting to note that, despite statistical variations, likely impacted by the small sample sizes in both groups, the mean differences and effect sizes are larger for every metric of skeletal muscle in the passive hot-water group compared to the control. These data will be useful for future studies looking to see if non-exhaustive skeletal muscle tests can differentiate between different training strategies.

## 5. Conclusions

Six weeks of HIIT improved classic markers of endurance training from cardiopulmonary exercise testing. Moreover, non-invasive and non-exhaustive tests of skeletal muscle hemodynamics all improved alongside improved aerobic capacity. These data imply that NIRS kinetics can be monitored during and after limb occlusion and/or steady-state exercise to monitor skeletal muscle adaptation.

## Figures and Tables

**Table 1:** Characteristics of participants.

Characteristic	Total (*n* = 18, 8 Female, 10 Male)
Age (year)	27.9 ± 5.3
Stature (cm)	176.8 ± 7.7
Body mass (kg)	75.7 ± 13.6
BMI (kg/m^2^)	24.1 ± 3.1
Systolic blood pressure (mmHg)	130 ± 17.3
Diastolic blood pressure (mmHg)	80 ± 9.4
Resting HR (bpm)	74 ± 7.9

BMI, body mass index; HR, heart rate.

**Table 2:** Summary changes in maximum aerobic capacity pre- and post-HIIT.

Parameter	Incremental Test (*n* = 16)			
	Pre	Post	*d*	*p* Value
**_abs_** ***V*** **O_2max_ (L·min^−1^)**	3.60 ± 0.67	4.03 ± 0.66	0.65	<0.001 *
**_rel_** ***V*** **O_2max_ (mL·kg·min^−1^)**	47.69 ± 9.49	53.56 ± 8.29	0.66	<0.001 *
**HR_max_ (bpm)**	186 ± 7.92	183 ± 7.11	0.35	0.009 *
**PPO (w)**	300 ± 47.1	320 ± 48.9	0.41	<0.001 *

HIIT = high-intensity interval training; _abs_*V*O_2max_ = absolute maximum oxygen uptake; _rel_*V*O_2max_ = relative maximum oxygen uptake; HR_max_ = maximum heart rate; PPO = peak power output; *d* = effect size (Cohen’s d). * Significantly different (*p* < 0.05).

**Table 3:** Summary changes pre- and post-HIIT.

Parameter	Pre	Post	*d*	*p* Value
**Cuff occlusion test**				
**Baseline sFBF (mL·min^−1^)**	66.91 ± 22.9	77.08 ± 30.8	0.33	0.13
**Baseline SmO_2_ (%)**	64.58 ± 7.26	64.61 ± 4.21	0.005	0.99
**SSE**				
**_abs_** ***V*O_2_ (L·min^−1^)**	2.66 ± 0.46	2.50 ± 0.43	0.37	0.006 *
**_rel_** ***V*O_2_ (mL·kg·min^−1^)**	35.1 ± 5.13	33.3 ± 5.07	0.35	0.009 *
**Exercise HR (bpm)**	149 ± 9.29	142 ± 9.25	0.67	0.001 *
**PO (w)**	179 ± 27.7		-	-
**Baseline SmO_2_ (%)**	68.2 ± 5.03	67.2 ± 5.71	0.02	0.25
**Pre-recovery SmO_2_ (%)**	60.4 ± 7.05	63.4 ± 6.93	0.43	0.02 *

HIIT = high-intensity interval training; SSE = steady-state exercise; _abs_*V*O_2_ = absolute oxygen uptake; _rel_*V*O_2_ = relative oxygen uptake; HR = heart rate; PO = power output; sFBF, superficial femoral blood flow; *d* = effect size (Cohen’s d). * Significantly different (*p* < 0.05).

## Data Availability

The raw data supporting the conclusions of this article will be made available by the authors without undue reservation.

## Supplementary Materials

The following supporting information can be downloaded at: https://www.mdpi.com/article/10.3390/s26103167/s1, Tabel S1. Changes pre and Post HIIT in post occlusive vasodilator capacity control group (n = 8) and heat water group (n = 8), during and post occlusive SmO2 control group (n = 7) and heat water group (n = 5), post SSE SmO2 control group (n = 8) and heat water group (n = 5). Tabel S2. Changes pre and Post HIIT in maximum aerobic capacity control group (n = 8) and heat water group (n = 8). Figure S1. Changes in post occlusive skeletal muscle maximal vasodilator capacity pre and post 6 weeks of high intensity interval training (HIIT). Figure S2. Changes in during and post occlusive SmO2 pre and post 6 weeks of high intensity interval training (HIIT). Figure S3. Changes in post steady state exercise (SSE) SmO2 pre and post 6 weeks of high intensity interval training (HIIT).
