# Supplementary material for: Skeletal Muscle Blood Flow and NIRS Oxygenation Kinetics as a Tool to Evaluate Adaptations to High-Intensity Exercise Training
Source: Sensors (Basel). 2026 May 16;26(10):3167. doi: 10.3390/s26103167 (PMC13210578; doi:10.3390/s26103167)
Supplement: Supplementary file 1 [file sensors-26-03167-s001.zip › sensors-4275214-supplementary.pdf]

Tabel S1. Changes pre and Post HIIT in post occlusive vasodilator capacity control group (n=8) and heat water group (n=8), during and post occlusive SmO_2 control group (n=7) and heat water group (n=5), post SSE SmO_2 control group (n=8) and heat water group (n=5)

	**HIIT**					**HIIT+Heat**				
	Pre	Post	Mean difference	P-value	*d*	Pre	Post	Mean difference	P-value	*d*
Reactive hyperemia										
**RH peak blood flow (ml·min^{-1})**	1390 (415)	1455 (320)	65.01 (242.9)	0.47	0.18	1256 (394)	1556 (370)	299.5 (296.1)	0.02*	0.78
**ΔsFBF baseline-peak (ml·min^{-1})**	1314 (401)	1370 (337)	56.21 (257.7)	0.55	0.15	1192 (382)	1473 (345)	281.0 (299.9)	0.03*	0.77
During and post occlusive SmO_2										
**Slope Occlusion (%·s^{-1})**	-0.057 (0.025)	-0.077 (0.036)	-0.020 (0.023)	0.06	0.62	-0.059 (0.015)	-0.062 (0.013)	-0.003 (0.007)	0.42	0.23
**Rep 10s (%·s^{-1})**	0.79 (0.34)	1.14 (0.54)	0.35 (0.28)	0.01*	0.78	0.69 (0.19)	1.23 (0.33)	0.53 (0.30)	0.01*	1.95
**R_{peak} (%·s^{-1})**	1.18 (0.52)	1.67 (0.85)	0.48 (0.64)	0.09	0.69	1.14 (0.27)	1.70 (0.54)	0.55 (0.34)	0.02*	1.31
Post SSE SmO_2										
**Rep 10s (%·s^{-1})**	0.31 (0.23)	0.26 (0.24)	-0.045 (0.073)	0.12	0.21	0.23 (0.14)	0.15 (0.10)	-0.075 (0.073)	0.08	0.62
**R_{bl} (%·s^{-1})**	0.46 (0.39)	0.40 (0.36)	-0.055 (0.108)	0.19	0.15	0.34 (0.20)	0.22 (0.10)	-0.119 (0.148)	0.14	0.73

HIIT= high-intensity interval training; SSE= steady state exercise; sFBF= superficial femoral blood flow; RH= reactive hyperemia; Rep 10s = the muscle reoxygenation rate over 10 seconds; R_{Peak}= the relative muscle oxygenation to peak; R_{bl}= relative rate of muscle reoxygenation back to base line; *d* = effect size (Cohen's d).

Tabel S2. Changes pre and Post HIIT in maximum aerobic capacity control group (n=8) and heat water group (n=8)

	HIIT					HIIT+Heat				
	Pre	Post	P-value	Mean difference	*d*	Pre	Post	P-value	Mean difference	*d*
**$_{abs}\dot{V}O_{2max}$ (L·min-^{1})**	3.73 (0.82)	4.12 (0.81)	0.01*	0.37 (0.33)	0.47	3.47 (0.49)	3.95 (0.53)	0.01*	0.48 (0.41)	0.95
**$_{rel}\dot{V}O_{2max}$ (ml·kg·min^{-1})**	48.46 (10.50))	53.97 (10.18)	0.01*	5.5 (4.6)	0.53	46.93 (9.03)	53.15 (6.60)	0.01*	6.22 (5.0)	0.78
**HRmax (bpm)**	186 (9.19)	184 (7.61)	0.09	-2.11 (3.04)	0.24	185 (7.01)	182 (6.90)	0.07	-3.12 (4.04)	0.43
**PPO (w)**	309 (51.39)	325 (52.06)	0.008*	16.88 (13.08)	0.33	292 (44.07)	312 (48.03)	<0.001*	20.63 (7.28)	0.46

HIIT= high-intensity interval training; $_{abs}\dot{V}O_{2max}$= absolute maximum oxygen uptake; $_{rel}\dot{V}O_{2max}$= relative maximum oxygen uptake; HR_{max}= maximum heart rate; PPO= peak power output; *d*= effect size (Cohen's d).

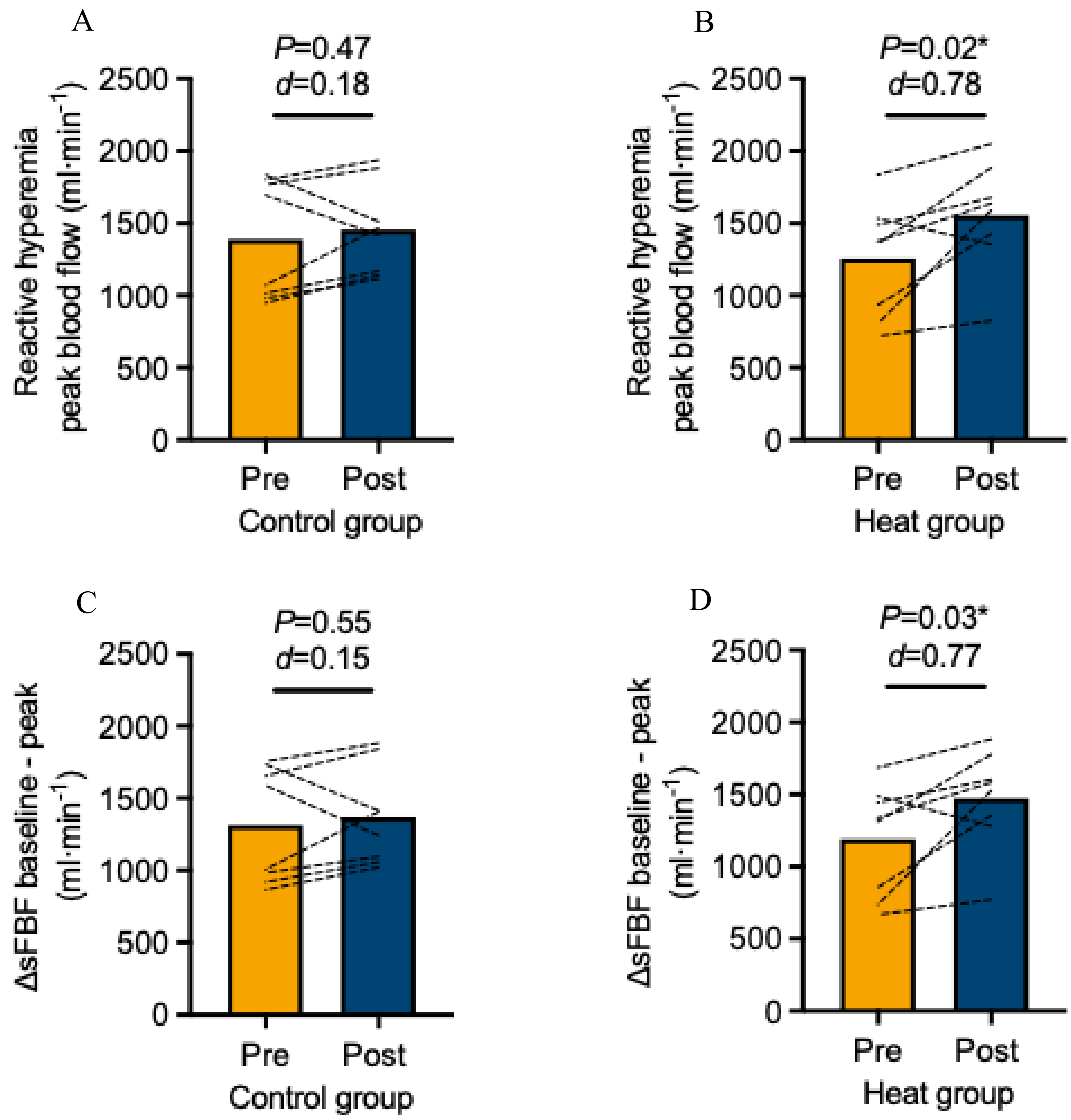

Figure S1. Changes in post occlusive skeletal muscle maximal vasodilator capacity pre and post 6 weeks of high intensity interval training (HIIT). Peak blood flow A) control group (n=8) and B) heat water group (n=8), the difference of peak superficial femoral blood flow (sFBF) and baseline blood flow C) control group and D) heat water group

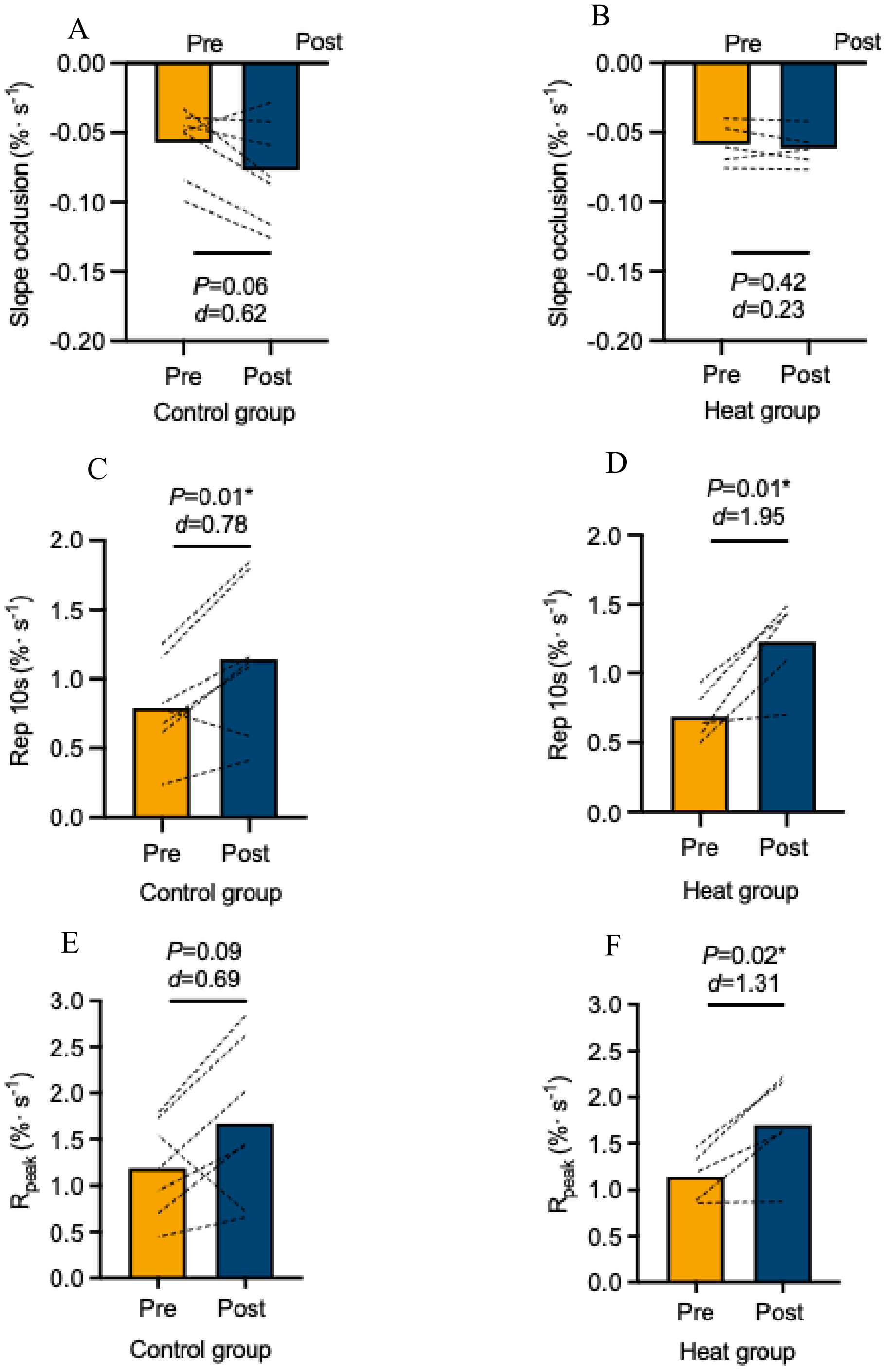

Figure S2. Changes in during and post occlusive SmO_2 pre and post 6 weeks of high intensity interval training (HIIT). The oxygenation occlusion A) control group (n=7) and B) heat water group (n=5), the muscle reoxygenation rate over 10 seconds (Rep 10s) C) control group and D) heat water group, the relative muscle oxygenation to peak (R_{Peak}) E) control group and F) heat water group.

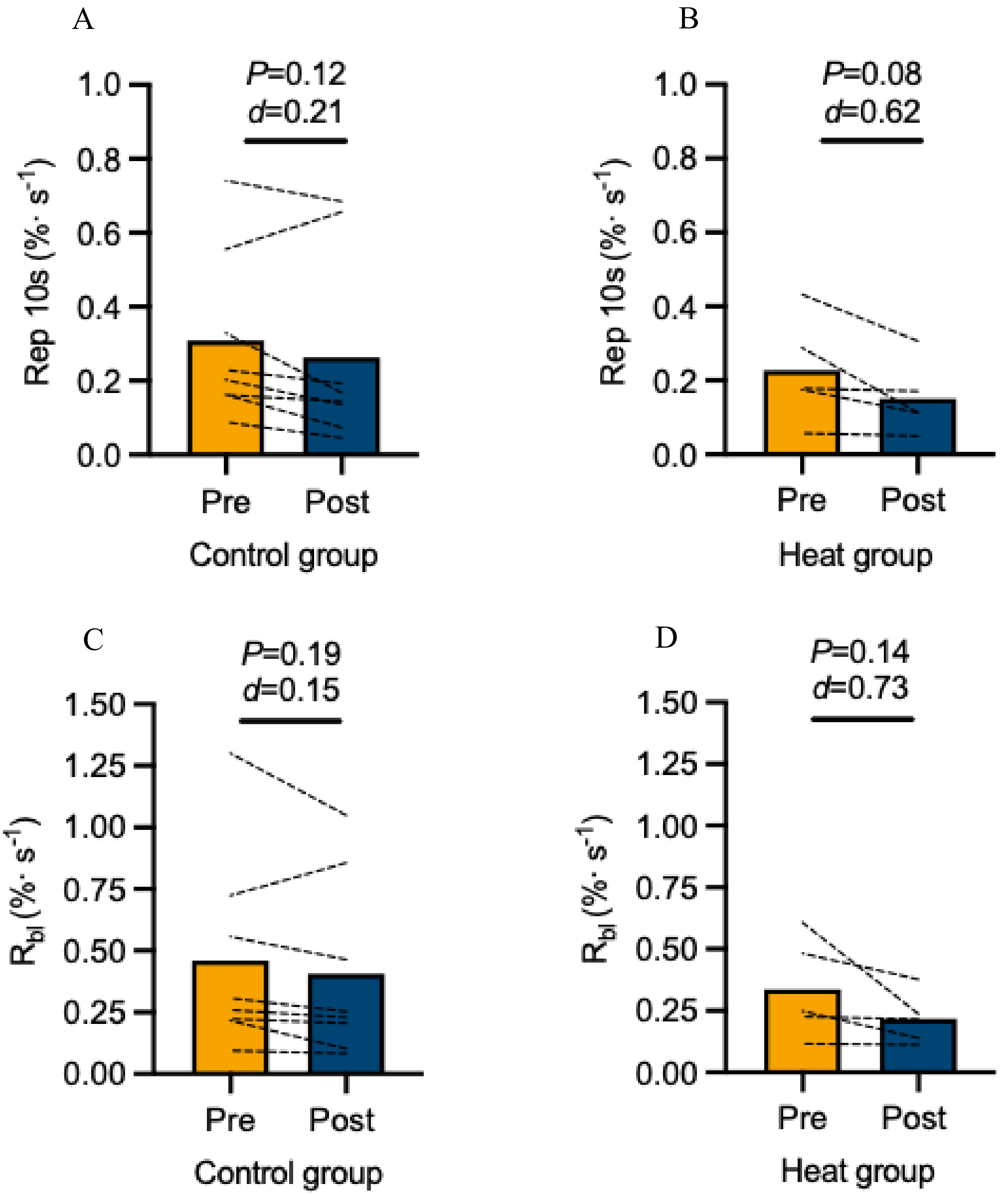

Figure S3. Changes in post steady state exercise (SSE) SmO_2 pre and post 6 weeks of high intensity interval training (HIIT). the muscle reoxygenation rate over 10 seconds (Rep 10s) A) control group (n=8) and B) heat water group (n=5), relative rate of muscle reoxygenation back to base line (R_{bl}) C) control group and D) heat water group.
